# Perspectives to Performance of Environment and Health Assessments and Models—From Outputs to Outcomes?

**DOI:** 10.3390/ijerph10072621

**Published:** 2013-06-26

**Authors:** Mikko V. Pohjola, Pasi Pohjola, Marko Tainio, Jouni T. Tuomisto

**Affiliations:** 1 National Institute for Health and Welfare, Department of Environmental Health, P.O. Box 95, Kuopio FI-70701, Finland; E-Mail: jouni.tuomisto@thl.fi; 2 Nordem Oy, Jynkänmäenkatu 1 D 1, Kuopio 70840, Finland; 3 National Institute for Health and Welfare, Service System Department, P.O. Box 30, Helsinki FI-00271, Finland; E-Mail: pasi.pohjola@thl.fi; 4 Systems Research Institute, Polish Academy of Sciences, Newelska 6, Warszawa 01-447, Poland; E-Mail: marko.tainio@ibspan.waw.pl

**Keywords:** assessment, model, evaluation, environment, health, performance, management, quality, uncertainty, effectiveness

## Abstract

The calls for knowledge-based policy and policy-relevant research invoke a need to evaluate and manage environment and health assessments and models according to their societal outcomes. This review explores how well the existing approaches to assessment and model performance serve this need. The perspectives to assessment and model performance in the scientific literature can be called: (1) quality assurance/control, (2) uncertainty analysis, (3) technical assessment of models, (4) effectiveness and (5) other perspectives, according to what is primarily seen to constitute the goodness of assessments and models. The categorization is not strict and methods, tools and frameworks in different perspectives may overlap. However, altogether it seems that most approaches to assessment and model performance are relatively narrow in their scope. The focus in most approaches is on the outputs and making of assessments and models. Practical application of the outputs and the consequential outcomes are often left unaddressed. It appears that more comprehensive approaches that combine the essential characteristics of different perspectives are needed. This necessitates a better account of the mechanisms of collective knowledge creation and the relations between knowledge and practical action. Some new approaches to assessment, modeling and their evaluation and management span the chain from knowledge creation to societal outcomes, but the complexity of evaluating societal outcomes remains a challenge.

## 1. Introduction

Environment and health assessments and models are to change the world [1], and not only the world of researchers, assessors, and modelers. Rather, they should have effect on the decisions and actions that influence the environment we live in. The societal impacts of assessments and models should not be evaluated merely in terms of scientific quality of their outputs and their so called process effects [2] in the social context of researchers, assessors, and modelers. The performance of assessments and models need to be evaluated also in terms of their outcomes in the broader societal context of everyday life, *i.e.*, practical decisions and actions by policy makers, business managers as well as individual citizens.

Especially in a time when arguably knowledge-based policies and policy-relevance of research is called for more than ever before, there is an increasing need to evaluate the success of environment and health assessments and models according to their societal effectiveness. In a recent thematic issue on the assessment and evaluation of environmental models and software [3], Matthews *et al.* [2] suggested that the success of environmental modeling and software projects should be evaluated in terms of their outcomes, *i.e.*, changes to values, attitudes, and behavior outside the walls of the research organization, not just their outputs. However, until now, there has been limited appreciation within the environmental modeling and software community regarding the challenges of shifting the focus of evaluation from outputs to outcomes [2].

The situation in the domain of environment and health related assessments, such as integrated assessment [4], health impact assessment [5], risk assessment [6,7,8], chemical safety assessment [9], environmental impact assessment [10], and integrated environmental health impact assessment [11] appears to be similar. A recent study on the state of the art in environmental health assessment revealed that although most assessment approaches aim to influence the society, this is rarely manifested in the principles and practices of evaluating assessment performance [12].

The emphasis in the scientific discourses on evaluating assessments and models has been on rather scientific and technical aspects of evaluation within the research domain, and perspectives that address the impacts of assessments and models in broader societal contexts have emerged only quite recently and are still relatively rare (*cf*. [13]). Such evaluations are qualitatively different [2], which indicates a need to reconsider the criteria and frameworks for evaluating assessment and model performance. Furthermore, evaluation of assessments and models is not only a matter of judging how good an assessment or a model is, but it also guides their making and the use of their outputs (*cf*. what you measure is what you get (WYMIWYG) in [14]).

In evaluation of societal effectiveness, both assessments and models are considered as instances of science-based support to decision making upon issues relevant to environment and health. They should thus both help us to understand nature and health, but also help us to formulate goals and propose and implement policies to achieve them [15] Assessments always involve modeling of some kind, at least implicit conceptual models. Conversely, modeling is also often identified with assessment [16]. In addition, decision support systems, information support tools, integrated modeling frameworks and other software tools and information systems to assist in developing, running, and analyzing models are here perceived as integral parts of assessment and modeling (*cf*. [17]).

Assessments and models can be considered e.g., as diagnostic, prognostic, or summative according to the kinds of questions they address [11], ex-ante or ex-post according to their timing in relation to the activities being assessed [18], and regulatory or academic according to the contexts of their development and application [12]. They can also be developed, executed, and applied by many kinds of actors, e.g., consultants, federal agencies or academic researchers. However, assessments and models, as perceived here, should be clearly distinguished from purely curiosity-driven basic research, as well as ad hoc assessments, and assessments or models made only to justify predetermined decisions.

**Figure 1:**
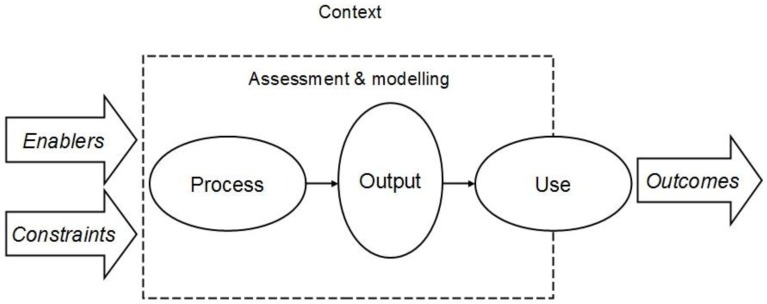
Assessment and modeling in interaction with their societal context (influenced e.g., by [2,19,20,21,22]).

Altogether, assessments and models can be considered as fundamentally having two purposes: (i) describing reality, and (ii) serving the needs of practical decision-making. Accordingly, the structure of the interaction between assessments and models and their societal context can be described as in Figure 1.

The endeavors of assessment and modeling, primarily located within the domain of researchers, assessors, and modelers (inside the dashed box in Figure 1), are here broken down into:

1. *Process*, the procedures and practices of assessment and modeling
2. *Output*, the assessment and model results and products, and
3. *Use*, the application of the assessment and model outputs

The surrounding context, containing policy making, business and everyday life at large, *enables*, and on the other hand also *constrains*, assessment and modeling, e.g., in the form of funding, facilities and education, but also as acceptance of or demand for assessments and models. The societal context is also the medium where the *outcomes* of assessments and models are realized. Use is located on the boundary between the assessment/modeling domain and the context, which indicates that the application of assessment and model outputs is the primary point of interaction between assessments or models and their societal context.

In following, the approaches to environment and health assessment and model performance in the scientific literature are reviewed and categorized according to which aspects are primarily seen to constitute the goodness of assessments and models. The review primarily focuses on methods, tools, and frameworks, which explicitly aim to identify the factors that determine the success of assessments and models and guide their evaluation. Therefore, methods, tools, and frameworks primarily aimed to support the execution of modeling, assessment or decisions processes are not emphasized. However, this distinction between supporting evaluation and execution is not always clear, but also in these cases, the review primarily focuses on the aspects related to identification and evaluation of the perceived factors of performance.

The identified perspectives to assessment and model performance are called: (i) quality assurance/control, (ii) uncertainty analysis, (iii) technical assessment of models, (iv) effectiveness, and (v) other perspectives. The question underlying this review is how much and how is the interaction with the societal context reflected upon in the approaches to environment and health assessment and model performance in contemporary scientific literature? The understanding of societal context is not limited to communities of experts dealing with assessments and models, but extends to include also policy making and practical actions influenced by or relevant to environment and health assessments and models. The purpose is thus not to discuss the details of different methods, tools and frameworks, but instead map how the approaches and their perceived factors of performance relate to the aspects of assessments and models in interaction with their societal context, as illustrated in Figure 1. Recent contributions in the literature are emphasized, but some important or illustrative examples that were published before 2000 have been included as well. After the review, the approaches and perspectives are discussed in terms of their capability to serve the needs of outcome oriented evaluation and management of assessments and models. In addition, a framework for developing more comprehensive outcome oriented approaches is proposed.

## 2. Perspectives to Assessment and Model Performance

### 2.1. Quality Assurance/Control

One of the major themes in assessment and model performance related literature can be referred to as quality assurance/control (QA/QC) perspective. The focus in this perspective is primarily on determining how the processes of assessment and modeling, sometimes also decision making, are to be conducted in order to assure the quality of the output.

There are multiple alternative definitions for quality (see e.g., [23]). However, as regards assessment and models, the interpretation is mostly analogous with the perception in the ISO-9000 framework, *i.e.*, as the organizational structures, responsibilities, procedures, processes, and resources to assure and improve quality [24]. In addition, the hierarchy of evidence in medical science, ranking types of evidence strictly according to the procedure by which they were obtained [25], is an example of the quality assurance/control perspective. However, as pointed out by Cartwright [26] with regard to randomized controlled trials, the procedure alone cannot guarantee delivery of useful information in practical contexts.

One common variation of this perspective is stepwise procedural guidance (Table 1). Such guidance provides relatively strict and detailed descriptions of the steps or phases of an assessment or modeling process that are to be executed in a more or less defined order. Faithful execution of the procedure is assumed to lead to good outputs. A similar, but often less rigorous, variation of the QA/QC perspective is check list guidance emphasizing issues that need to be taken account of in the assessment or modeling process or their evaluation. The checklists can be more or less detailed and they usually do not strictly define the order or sequence of execution.

In addition, the accounts that address evaluation of input quality can be considered as manifestations of the QA/QC perspective (Table 1). However, the primary focus in QA/QC is often on the outputs, and the input quality evaluations typically complement uncertainty analysis or technical assessments of models (see below). For example, model parameter uncertainty analysis can be considered as an example of evaluation of input quality, but in practice, it is most often considered as an aspect of either uncertainty analysis or technical assessment of models.

**Table 1:** Examples of quality assurance/control perspective to assessment and model performance.

Type	Description
Stepwise procedural guidance	Ten iterative steps in development and evaluation of environmental models [27]
	HarmoniQuA guidance for quality assurance in multidisciplinary model-based water management [28]
	Methodology for design and development of integrated models for policy support [29]
	Framework for integrated environmental health impact assessment [11]
	BRAFO tiered approach for benefit-risk assessment of foods [30]
	Generic framework for effective decision support through integrated modeling and scenario analysis [31]
	Formal framework for scenario development in support of environmental decision making [32]
Check list guidance	Seven attributes of good integrated assessment of climate change [33]
	List of end use independent process based considerations for integrated assessment [34]
	QA/QC performance measurement scheme for risk assessment in Canada [35]
	Check list for quality assistance in environmental modeling [36]
Evaluation of input quality	Pedigree analysis in model-based environmental assessment [37]
	Methodology for recording uncertainties about environmental data [38]
	Method for analyzing assumptions in model-based environmental assessments [39]

Characteristic for stepwise guidance is that it attempts to predetermine a procedure in order to guarantee good quality of outputs. As such, it takes a proactive approach to managing performance in anticipation of future needs. Checklist guidance and evaluation of input quality can also be applied proactively, but the examples found in literature mostly represent a reactive approach of evaluating already completed assessments and models.

The quality assurance/control perspective relates predominantly to the domain of experts, yet most of the approaches within the perspective also intend to reflect the needs of the broader societal context, particularly policy making. The evaluation and management of performance, however, mainly considers the aspects of making assessments and models. Correspondingly, most of the approaches within this perspective simultaneously both identify factors of assessment and model performance and provide guidance for execution of assessment and modeling processes.

### 2.2. Uncertainty Analysis

Another major theme in the assessment and model performance literature is the uncertainty analysis perspective. The contributions within this perspective vary significantly, ranging from descriptions of single methods to overarching frameworks, but the common idea is characterization of certain properties of the assessment and model outputs. Fundamentally, the perspective builds on quantitative statistical methods based on probability calculus [40], but also other than probability-based approaches to uncertainty have been presented [41,42]. However, the non-probabilistic approaches can be considered as mostly complementary, not competitive, to the probabilistic approaches [43]. Many manifestations of this perspective in the context of environment and health assessment and models also extend to consider qualitative properties of the outputs.

**Table 2:** Examples of uncertainty analysis perspective to assessment and model performance.

Type	Description
Identification of kinds of uncertainty	Conceptual basis for uncertainty management in model-based decision support [44]
	Uncertainty in epidemiology and health risk and impact assessment [45]
	Uncertainty in integrated assessment modeling [46]
Guidance on dealing with uncertainties	Knowledge quality assessment for complex policy decisions [47]
	Operationalizing uncertainty in integrated water resource management [48]
	Framework for dealing with uncertainty in environmental modeling [49]
Methods for uncertainty analysis	Approaches for performing uncertainty analysis in large-scale energy/economic policy models [50]
	Modeling of risk and uncertainty underlying the cost and effectiveness of water quality measures [51]
	Addressing uncertainty in decision making supported by Life Cycle Assessment [52]
	Sensitivity analysis of model outputs with input constraints [53]

One variation of the uncertainty analysis perspective is identification of the kinds and sources of uncertainty in assessment and model outputs (Table 2). Some uncertainties are often considered as being primarily expressible in quantitative, while others in qualitative terms. The sources of uncertainty may include aspects of the assessment and modeling processes, and in some cases also intended or possible uses and use contexts of the outputs are acknowledged.

Also guidance on how to assess or deal with different kinds of uncertainties exist (Table 2). Such frameworks usually combine qualitative and quantitative aspects of uncertainty deriving from various sources. Consequently, aspects of the assessment and modeling processes, e.g., input quality and user acceptance, are often also included in the frameworks. The primary focus still remains in the characteristics of the assessment and model.

Numerous more or less explicit methods, means and practices to analyze uncertainties of assessment and model outputs also exist (Table 2). In addition to the standard statistical characterization, for example sensitivity, importance, and value of information analysis and Bayesian modeling are essential in the context of environment and health assessment and models. Such methods are dominantly quantitative.

In the uncertainty analysis perspective, it appears typical that the issue of uncertainty is approached from an external observer’s point of view. The evaluation of performance is thus mainly considered as a separate, reactive activity taking place in addition to the actual assessment or modeling process, not as its integral proactive part. The evaluation usually takes place within the expert domain and primarily serves the purpose of describing reality, and only indirectly serves the secondary needs of the broader societal context. The approaches to uncertainty primarily focus on the assessment or model results, and mostly do not provide direct guidance on how to conduct modeling, assessment or decision processes.

### 2.3. Technical Assessment of Models

This perspective focusing on characteristics of models is particularly present in the modeling literature. In addition, different kinds of software tools that are applied in developing, running, and analyzing models can be evaluated similarly as models.

The object of interest in the technical assessment of models is development and application of formal methods for testing and evaluating models within defined domains of application (Table 3). Generally, model evaluation and performance is considered to cover structural features of models, representativeness of model results in relation to a certain part of reality, as well as usefulness with regard to a designated task (*cf*. [54]). However, usefulness mainly refers to expert use of models, corresponding mostly to the so-called process effects, *i.e.*, changes in the capacity of those engaged in the modeling and assessment endeavors, rather than outcomes (*cf*. [2]). Most commonly, technical assessment of models takes place in terms of validation and verification by comparing models and their results against each other or against measured data (e.g., [55,56]), although it has also been argued that models are fundamentally non-validatable [57].

A variation of this perspective, more common for the discourses in assessment literature, is analysis of model uncertainty (Table 3). Here the aim typically is to characterize the properties of a model in order to be able to correctly interpret or evaluate its outputs. Model uncertainty is often considered as one aspect of a broader uncertainty analysis concept.

The technical assessment of models is predominantly reactive, as it requires an existing model or software system that can be tested and analyzed. The evaluation, however, is usually perceived as an integral part of the model development, not a separate entity, enabling application of technical assessment of models in different developmental stages within the modeling or assessment process. On the other hand, the common practice of self-evaluation of models may also lead to e.g., limited usability, credibility and acceptability due to lack of interaction with the broader societal context. However, some approaches extend to explicitly take account of the needs of e.g., policy making and engage the model users in the evaluation. Somewhat comparably to uncertainty analysis, the approaches in this perspective focus on the model as the result of a modeling process. However, the evaluations are often also intended and applied as guidance to the execution of modeling processes.

**Table 3:** Examples of technical assessment of models perspective to assessment and model performance.

Type	Description
Means for model and software evaluation	Success factors for integrated spatial decision support systems [58]
	Criteria for environmental model and software evaluation [59]
	Terminology and methodological framework for modeling and model evaluation [60]
	Evaluation methods of environmental modeling and software in a comprehensive conceptual framework [2]
	Top-down framework for watershed model evaluation and selection [61]
	Overview of atmospheric model evaluation tool (AMET) [62]
	Appropriateness framework for the Dutch Meuse decision support system [63]
	Empirical evaluation of decision support systems [64]
	Numerical and visual evaluation of hydrological and environmental models [65]
Evaluation of models	Evaluating an ecosystem model for wheat-maize cropping system in North China [66]
	Parameterization and evaluation of a Bayesian network for use in an ecological risk assessment [67]
	Evaluation of quantitative and qualitative models for water erosion assessment in Ethiopia [68]
	Evaluation of modeling techniques for forest site productivity prediction using SMAA [69]
Analysis of model uncertainty	Model uncertainty in the context of risk analysis [70]
	Scenario, model and parameter uncertainty in risk assessment [71]
	Framework for dealing with uncertainty due to model structure error [72]

### 2.4. Effectiveness

Whereas the three former perspectives can be considered conventional, emphasis of assessment and model effectiveness has become a major topic only recently in the assessment and model performance related literature.

In the effectiveness perspective, the aim of assessments and models is perceived as promotion of changes in values, attitudes, and behavior outside the walls of the research community (*cf*. [2]) by maximizing the likelihood of an assessment process to achieve the desired results and the goals set for it [73]. In principle, here assessment and model performance is thus determined by the impacts delivered into the broader societal context. However, as it might take years to achieve set goals and it often is not immediately clear whether an observed change is a result of a specific decision or action, evaluation of outcomes is often perceived as very difficult, if not impossible [74], and possibly even leading to incorrect conclusions regarding effectiveness (*cf*. [75]). Consequently, the effectiveness criteria and frameworks (Table 4) often address aspects of process and output, as well as contextual enablers and constraints, rather than outcomes, as determinants of effectiveness. Some contributions also make a distinction between (immediate) impacts and (indirect) outcomes. As a result, although the aim is to address the outcomes, some approaches to effectiveness resemble the checklist guidance of quality assurance/control (see Table 1).

**Table 4:** Examples of effectiveness perspective to assessment and model performance.

Type	Description
Frameworks and criteria for effectiveness	Framework for the effectiveness of prospective human impact assessment [74]
	Process, impact and outcome indicators for evaluating health impact assessment [76]
	Criteria for appraisal of scientific inquiries with policy implications [77]
	Necessary conditions and facilitating factors for effectiveness in strategic environmental assessment [78]
	Components of policy effectiveness in participatory environmental assessment [79]
	Dimensions of openness for analyzing the potential for effectiveness in participatory policy support [80]
	Properties of good assessment for evaluating effectiveness of assessments [81]
Effectiveness evaluations	Several cases of evaluating effectiveness of health impact assessment in Europe [82]
	General effectiveness criteria for strategic environmental assessment and their adaptation for Italy [83]
	Environmental impact assessment evaluation model and its application in Taiwan [84]
	Effectiveness of the Finnish environmental impact assessment system [85]
	Example of outcome evaluation for environmental modeling and software [2]
Use of models, tools and outputs	User interaction during development of a decision support system [86]
	Review of factors influencing use and usefulness of information systems [87]
	Bottlenecks of widespread usage of planning support systems [88]
	Framework to assist decision makers in the use of ecosystem model predictions [89]
	Analysis of contribution of land-use modeling to societal problem solving [90]
	Use of decision and information support tools in desertification policy and management [91]
	Developing tools to support environmental management and policy [92]
	Role of computer modeling in participatory integrated assessments [93]
	Usage and perceived effectiveness of decision support systems in participatory planning [94]
	Credible uses of the distributed interactive simulation (DIS) system [95]
	Analysis of interaction between environmental health assessment and policy [12]

The approaches emphasizing the use of models, tools and their outputs can also be considered as a manifestation of the effectiveness perspective (Table 4). They can generally be characterized as attempts to operationalize the interaction between assessments or models and the practical uses of their outputs. Most of the contributions are, however, relatively tool-centered, and most often little attention is given to the cognitive processes involved in the delivery and reception of information produced by assessments and models.

Correspondingly, the effectiveness perspective clearly intends to serve the needs of the broader societal context. However, due to practical challenges of measuring societal changes, the focus easily shifts towards guidance of assessment and modeling within the expert domain. Comparably to quality assurance/control, many approaches in this perspective intertwine the principles for determining performance with the guidance for executing assessment, modeling, and decision processes.

### 2.5. Other Perspectives

Many contributions to assessment and model performance in relevant literature can be quite comfortably located within the four perspectives above. However, there are also some other approaches addressing information quality, acceptance and credibility, communication, participation, and decision process facilitation (Table 5) that deserve mentioning.

Assessment and modeling are essentially processes of producing structured information. Therefore, the contributions regarding information quality, even outside the fields of assessment and modeling, are of relevance here. Like the uncertainty analysis perspective, information quality looks into certain properties of an information product. Similarly, the variation among contributions addressing information quality is big.

Credibility is often considered necessary for acceptance of assessment and modeling endeavors and their outputs. It can be obtained more or less formally or informally e.g., through peer review, extended peer-review [96] or reputation of the participants involved in assessment and modeling. Acceptance and credibility are often considered as aspects of broader frameworks along with other determinants of performance.

In addition, communication of results, e.g., in terms of communicating uncertainties and risk information, relates to assessment and model performance. However, the issues of communication are often not considered as integral parts of modeling and assessments endeavors. For example, risk assessment, risk management and risk communication are traditionally considered as separate, yet interrelated, entities, each having their own aims, practices, and practitioners (e.g., [97]).

Techniques for involving stakeholders and public most often do not scrutinize performance of assessments and models, but at least implicitly determine certain factors of their performance while guiding conducting of participatory processes. Participation can relate to assessment and modeling or decision making.

There are also several approaches to facilitating decision processes deriving from the domains of decision analysis, operations research and management science in general. Rather than approaches to evaluating assessment and model performance, they are particularly intended for framing and structuring problems as well as guiding the decision processes in searching solutions to them. In some approaches, explicit success criteria for decision processes are presented and some approaches are also applicable as tools for social knowledge creation in stakeholder involvement.

The approaches mentioned as other perspectives vary in their relation to the broader societal context. Many of them, however, have a connection to issues regarding how assessments and models are perceived and interpreted outside the expert domain. Whereas information quality focuses on characteristics of an information product, the rest of the approaches in this category primarily aim to determine and guide procedures of assessment and modeling, communication, participation, or decision making in order to promote certain aspects of assessment, model or decision performance.

**Table 5:** Examples of other perspectives to assessment and model performance.

Type	Description
Information quality	A conceptual framework of data quality [98]
	An asset valuation approach to value of information [99]
	Ten aspects that add value to information [100]
	Knowledge quality in knowledge management systems [101]
Acceptance and credibility	Obtaining model credibility through peer-reviewed publication process [58]
	Model credibility in the context of policy appraisal [102]
	Salience, credibility and legitimacy of assessments [103]
Communication	Uncertainty communication in environmental assessments [104]
	Check list for assessing and communicating uncertainties [105]
	Communication challenges posed by release of a pathogen in an urban setting [106]
	Clarity in knowledge communication [107]
Participation	Openness in participation, assessment and policy making [80]
	Purposes for participation in environmental impact assessment [108]
	OECD/NEA stakeholder involvement techniques [109]
	Participation guide for the Netherlands Environmental Assessment Agency [110]
Decision process facilitation	Rational analysis for a problematic world [111]
	Brief presentations of numerous decision support tools (website) [112]
	Decision analysis as tool to support analytical reasoning [113]

Although the approaches in this category relate to how assessments and models may influence the broader societal context, e.g., through societal decision making and social learning, most of them are not strongly linked to the perspectives to assessment and model performance described above. Instead, many approaches seem to be addressing a certain relevant, but not integral, entity related to assessments and models in interaction with the broader societal context*.*

## 3. Discussion

### 3.1. Overview of Approaches and Perspectives

It seems that none of the perspectives reviewed above or any individual approaches alone sufficiently serve the needs of outcome oriented evaluation and management of assessment and model performance. In most approaches, the main emphasis is on the processes and outputs of assessment and modeling, and correspondingly primarily addressing the needs from within the expert domain. Use, outcomes and other aspects of the broader societal context are addressed to a lesser extent, although more frequently in recent literature. Although the approaches focusing on processes and outputs may be robust, they tend to miss important aspects of interaction between assessments and models and the broader societal context. On the other hand, the approaches focusing on the interaction may be vaguer and still provide only partial solutions to considering how and why assessments and models influence their societal contexts, particularly societal decision making. Altogether, the needs of the broader societal context are, although to varying degrees, recognized in most perspectives to assessment and model performance. However, explicit measurement and treatment of outcomes in the broader societal context is difficult.

Certain illuminating differences similarities can be identified between the perspectives and approaches described above. These relate e.g., to which parts of the chain from knowledge creation to outcomes (see Figure 1) are addressed, whether the approach considers products or processes of assessment, modeling and decision making, and whether evaluation is perceived as a separate entity or intertwined with guidance of executing assessment, modeling or decision processes. 

Uncertainty analysis and technical assessment of models are somewhat similar in the sense that they focus on the products of assessment and modeling. Uncertainty analysis is, however, more clearly a separate and often reactive process, while technical assessment of models is often linked also to the execution of modeling processes. Both perspectives are mostly confined to the expert domain of assessors and modelers.

Quality assurance/control and effectiveness perspectives are similar in the sense that they often merge evaluation and guidance of processes, although some effectiveness approaches actually aim to consider outcomes, the products of decision making. However, as this is difficult, in practice the main difference between these perspectives is that whereas quality assurance/control focuses on assessment and modeling processes with little reference to the use processes they relate to, effectiveness perspective particularly attempts to look into the use of assessments and models in decision making.

Of the other considered perspectives, information quality is in principle quite similar to some uncertainty analysis approaches, although the focus of evaluation is often other than assessment or model results. Acceptance and credibility, communication, participation, and decision process facilitation all primarily determine procedures related, but not integral, to assessments, models and their use. The linkages to the factors determining and realizing assessment and model performance are, however, often implicit or weak.

Most of the approaches that explicitly identify factors of environment and health assessment and model performance thus focus on processes and outputs of assessment and modeling. This can be considered to be in line with the fact that the issues of effectiveness and policy-relevance of assessments and models have become major topics only during the last decades (as can be seen e.g., by searching scientific article databases). As assessors, modelers and researchers more generally have been lacking requirements and incentives for effectiveness and policy-relevance (*cf*. [114]), correspondingly the practices, principles and methods of performance management and evaluation have not developed to address these issues. Instead, the impacts of assessments and models have mostly been considered mainly in terms of their process effects (*cf*. [2]) within the communities of assessors and modelers. However, virtually all assessment and modeling endeavors in the fields of environment and health are motivated, at least nominally, by the aim to influence societal decisions and actions. The need to evaluate and manage assessments and models according to their societal outcomes thus seems justified.

The other perspectives complement the conventional approaches to assessment and model performance by addressing different aspects of interaction of assessments and models with their broader societal context. However, mostly they do not link seamlessly to evaluation and management of assessment and model performance. Only in the relatively recently emerged effectiveness perspective, the use of assessments, models, and their results in decision processes is recognized as a crucial part for assessment and model performance.

### 3.2. Towards New Approaches

**Figure 2:**
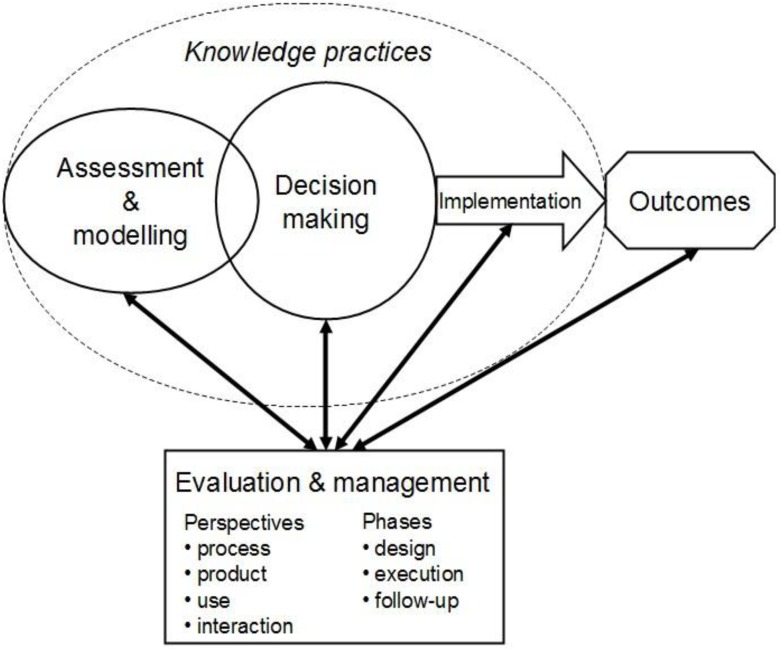
A framework for comprehensive evaluation and management of assessment and model performance. The chain from assessment and modeling to outcomes mostly consists of production, communication and application of knowledge in a societal context.

It appears that more comprehensive approaches that provide a better coverage of the different aspects of assessments and models in their societal context are needed to support evaluation and management of assessment and model performance. In practice, this requires taking account of the making of assessments and models, their use in decision making, practical implementation of the knowledge they deliver, as well as the consequential societal changes they evoke, *i.e.*, outcomes (Figure 2). Such approaches would combine the essential characteristics of the different perspectives reviewed above into one framework, methodology or combination of tools and provide both rigor and better linkage from evaluation and making of assessments and models to the outcomes.

However, a mere compilation of features taken from different perspectives would probably not be sufficient. A more thorough account of the mechanisms of collective knowledge creation and the relations between knowledge and action in a societal context is needed in order to bridge assessments and models with their outcomes [115]. Unfortunately, these aspects are barely even recognized in most current approaches to assessment and model performance.

The need to span the whole chain from knowledge creation to outcomes and bringing the producers and users of knowledge to a more intimate interaction for solving practical problems is recognized in some new approaches to assessment, modeling and their evaluation and management (e.g., [2,116,117]). The new approaches can be seen attempts to address the challenges of societal decision making which the more conventional approaches have not succeeded to resolve [12]. Most significantly the new approaches are different in the sense that they consider knowledge and knowledge-based action as their output, and software and information as means for their delivery. The pragmatic and socio-technical approaches also draw the attention to the practices of the collectives involving in intentional creation and use of knowledge in networks consisting of human actors and non-human objects (e.g., tools, models, information) mediating their interaction (see Figure 2 and [118]). Perceiving assessments and models interacting with their contexts as socio-material entities also promotes a multi-perspective approach to their evaluation (*cf*. [116]).

However, the complexity of evaluating the outcomes remains a challenge. In the eyes of an evaluator, the relative simplicity of considering only processes, outputs or direct impacts in tightly bound settings of expert activities may still appear inviting in comparison to attempting to account for complex indirect impacts within the broader social context. Unfortunately, this would not be adequate for serving the purposes of assessment, models and their evaluation.

In order to overcome this challenge, the new comprehensive approaches should not only focus on either processes, outputs, uses or outcomes of assessments and models, but particularly consider and address the knowledge that is created, transferred and applied within the intertwined processes of modeling, assessment and decision-making (see Figure 2). This means that the evaluation and management should be a continuous counterpart of designing and making assessments and models and applying their outputs in practice. After all, assessments and models can only be evaluated in relative terms, and their primary value is heuristic [119] Correspondingly, the use of assessments and models, as advocated by the effectiveness perspective, appears to be the most critical link in the chain from assessment and modeling to outcomes. The approaches to communication, participation and particularly decision process facilitation hold a lot of potential for developing the practices of evaluating and managing collective knowledge creation in decision making by means of assessments and models.

## 4. Conclusions

Altogether, the findings of the review can be briefly summarized as follows:

1. Conventional evaluation of assessments and models focuses on processes and outputs;
2. Recently also societal outcomes of assessments and models have been emphasized;
3. Effectiveness of assessments and models can be considered as their likelihood of delivering intended outcomes;
4. An outcome-oriented turn is taking place in assessment, modeling and their evaluation;
5. New approaches merge design, making and evaluation of assessments and models;
6. Assessments and models are useful means for facilitating collective knowledge creation e.g., in societal decision making.
